# Five miRNAs-mediated PIEZO2 downregulation, accompanied with activation of Hedgehog signaling pathway, predicts poor prognosis of breast cancer

**DOI:** 10.18632/aging.101934

**Published:** 2019-05-06

**Authors:** Weiyang Lou, Jingxing Liu, Bisha Ding, Luqi Jin, Liang Xu, Xia Li, Jing Chen, Weimin Fan

**Affiliations:** 1 Program of Innovative Cancer Therapeutics, Division of Hepatobiliary and Pancreatic Surgery, Department of Surgery, First Affiliated Hospital, College of Medicine, Zhejiang University, Key Laboratory of Combined Multi-Organ Transplantation, Ministry of Public Health, Key Laboratory of Organ Transplantation, Zhejiang Province, Hangzhou 313100, China; 2 Department of Intensive Care Unit, Changxing People’s Hospital of Zhejiang Province, Huzhou 313100, China; 3 First Affiliated Hospital of Jiaxing University, Zhejiang Province, Jiaxing 314000, China; 4 The National Education Base for Basic Medical Sciences, Zhejiang University School of Medicine, Zhejiang Province, Hangzhou 310058, China; * Equal contribution

**Keywords:** breast cancer, piezo-type mechanosensitive ion channel component 2 (PIEZO2), Hedgehog signaling pathway, prognosis, microRNA (miRNA)

## Abstract

Roles of Piezo-type mechanosensitive ion channel component 2 (PIEZO2) in cancer remain largely unknown. Herein, we explored PIEZO2 expression, prognosis and underlying mechanisms in cancer. Breast was selected as the candidate as its relatively higher expression level of PIEZO2 than other human tissues. Next, we identified a decreased expression of PIEZO2 in breast cancer compared with normal controls, and found that PIEZO2 expression positively correlated with estrogen receptor (ER) and progesterone receptor (PR) status but negatively correlated with human epidermal growth factor receptor 2 (HER2) status, Nottingham Prognostic Index (NPI) score, Scarff-Bloom-Richardson (SBR) grade, basal-like and triple-negative status. Subsequent analysis revealed that high expression of PIEZO2 had a favorable prognosis in breast cancer. 182 miRNAs were predicted to target PIEZO2. Among these miRNAs, five miRNAs (miR-130b-3p, miR-196a-5p, miR-301a-3p, miR-421 and miR-454-3p) possess the greatest potential in targeting PIEZO2. 109 co-expressed genes of PIEZO2 were identified. Pathway enrichment analysis showed that these genes were enriched in Hedgehog signaling pathway, including Cell adhesion molecule-related/downregulated by oncogenes (CDON). CDON expression was decreased in breast cancer and downregulation of CDON indicated a poor prognosis. Altogether, these findings suggest that decreased expression of PIEZO2 may be utilized as a prognostic biomarker of breast cancer.

## Introduction

With the rapid development of economy and aging of population, cancer has been one of the most prevalent and lethal diseases all over the world, which seriously threatens human health [1]. According to the statistics of the world cancer report, in 2014, approximately 14.1 million cancers were diagnosed and nearly 8.2 million cancer-related deaths occurred. The onset of cancer is an extremely complicated process, consisting of a series of steps. Multiple factors account for this severe situation, among which gene mutation is one cause closely linked to cancer occurrence and progression [2,3]. Although considerable improvements have been achieved in cancer diagnosis, therapy and prognosis over the past few years, the five-year survival rate of most cancer patients remains dismal [4]. The majority of cancer patients will eventually recur after current treatment. Therefore, establishing an effective prognostic biomarker can not only estimate prognosis of cancer patients but also predict therapeutic effect, thereby providing proper treatment and finally improving clinical outcomes.

Lately, microRNA (miRNA)-targeted genes have been demonstrated to function as predictors of prognosis in cancer patients. For example, Sheng *et al.* found that, loss of suppression of miR-206, kinesin family member 2A was significantly overexpressed in ovarian cancer and was associated with poor prognosis of patients with ovarian cancer [5]; Lei *et al.* suggested that miR-222-mediated downregulation of matrix metalloproteinase inhibitor 3 indicated a good prognosis for non-small cell lung cancer [6]. Piezo-type mechanosensitive ion channel component 2 (PIEZO2), a mechanically activated ion channel, has entered the eyes of researchers and scholars for few years. PIEZO2 belongs to the PIEZO family which are large transmembrane proteins with predicted transmembrane domains between 24 and 36 [7]. PIEZO2 is also an essential component of distinct mechanically-activated cation channels and has been found to play a key role in rapid adapting mechanically activated currents in somatosensory neurons. PIEZO2 dysregulation has been well documented to cause several diseases, such as Gordon syndrome, Marden-Walker syndrome and Arthrogryposis [8]. Recently, some studies have also suggested that aberrant expression of PIEZO2 is involved in cancer onset and progression [9–11]. However, previous studies regarding the roles of PIEZO2 in cancer and the underlying mechanisms how PIEZO2 exerts its impact on cancer are still insufficient and need to be further elucidated. Furthermore, the expression and prognostic role of PIEZO2 in human cancers, to date, have also not been fully determined. In this study, we first detected the expression of PIEZO2 in all types of cancer, especially in breast cancer. Then, the prognostic roles of PIEZO2 in breast cancer based on different clinicopathological features were assessed. Finally, we explored the underlying regulatory mechanisms of PIEZO2 in breast cancer.

## RESULTS

### Expression profile of PIEZO2 in human normal and cancer tissues

**Figure 1:**
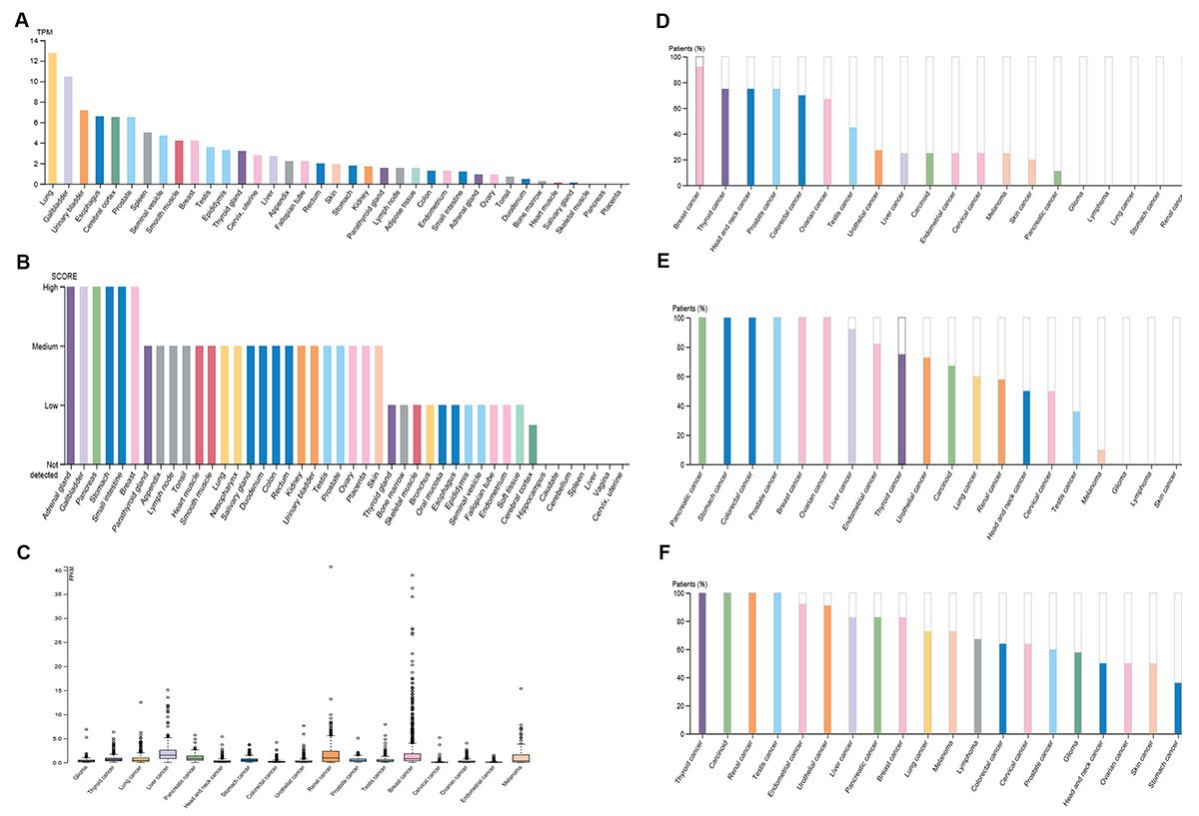
**Expression of PIEZO2 in normal and cancer tissues from the HPA database.** (**A**) PIEZO2 mRNA expression in different normal tissues; (**B**) PIEZO2 protein expression in different normal tissues; (**C**) PIEZO2 mRNA expression in different cancer tissues; (**D**) PIEZO2 protein expression in different cancer tissues (HPA031974); (**E**) PIEZO2 protein expression in different cancer tissues (HPA040616); (**F**) PIEZO2 protein expression in different cancer tissues (HPA015986).

A high and detectable expression level of a gene is one of the most important traits for being a promising diagnostic or prognostic biomarker. Therefore, in the first place, we determined the expression of PIEZO2 in different normal tissues using the Human Protein Atlas (HPA) database. The results demonstrated that lung, gallbladder, urinary bladder, esophagus, cerebral cortex, prostate, spleen, seminal vesicle, smooth muscle and breast were the top ten normal tissues according to expression values of PIEZO2 mRNA (Figure 1A). The top ten tissues, sorted by expression levels of PIEZO2 protein, were adrenal gland, gallbladder, pancreas, stomach, small intestine, breast, parathyroid gland, appendix, lymph node and tonsil (Figure 1B). Figure 1A and Figure 1B together told us that gallbladder and breast were the two proper candidates for further investigation. The expression of PIEZO2 mRNA and protein in different types of cancer was successively analyzed using the HPA database (Figure 1C-F). Among all types of cancer, breast cancer presented as the highest expression value of PIEZO2 in both mRNA and protein levels. Taken these findings together, breast was selected for further investigation.

### PIEZO2 expression is frequently decreased in breast cancer and inversely correlates with progression

**Figure 2:**
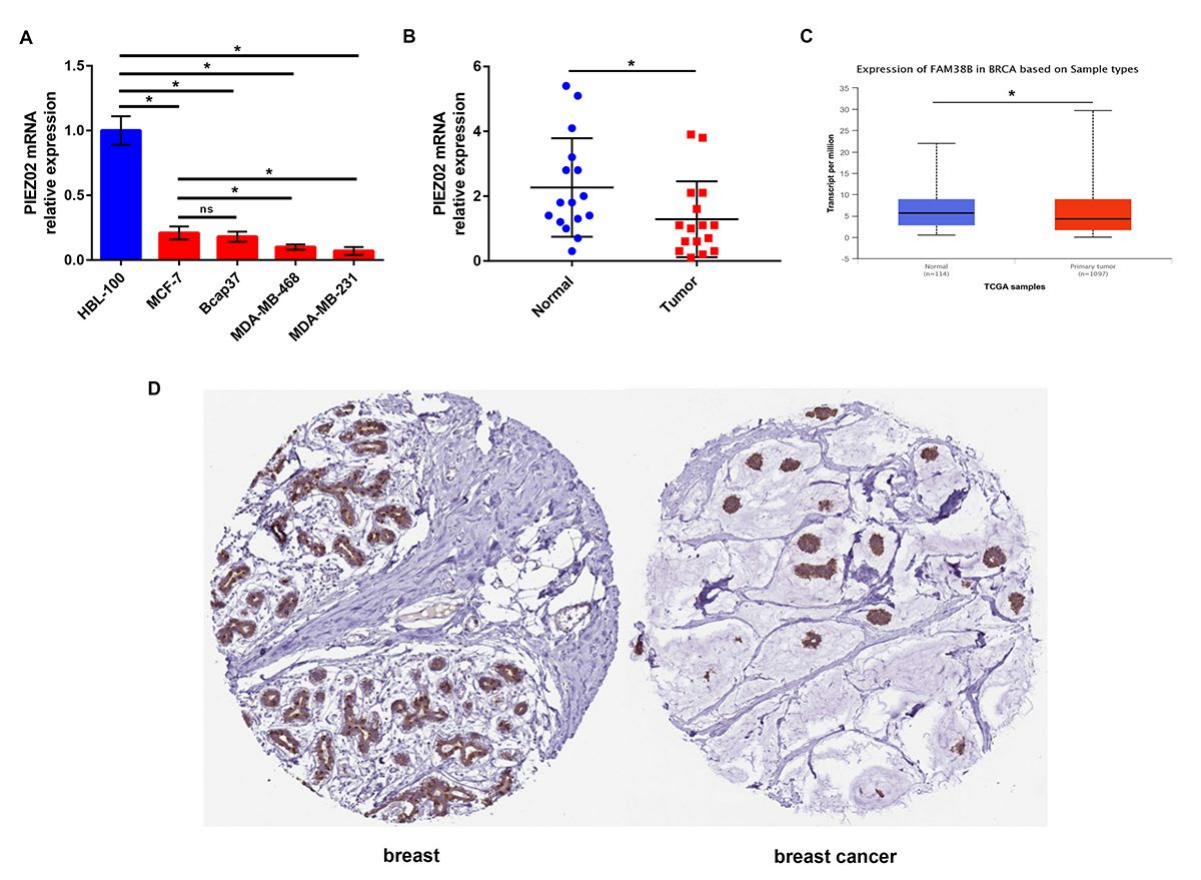
**Expression of PIEZO2 in breast cancer.** (**A**) PIEZO2 expression in breast cancer cell lines (MCF-7, Bcap37, MDA-MB-468 and MDA-MB-231) compared with that in normal breast cell line (HBL-100); (**B**) PIEZO2 expression in clinical breast cancer tissues compared with that in matched adjacent normal tissues (n=16); (**C**) expression of PIEZO2 (also known as FAM38B) in breast cancer compared with normal controls by analyzing UALCAN database; (**D**) PIEZO2 protein expression level in breast cancer tissue and normal breast tissue was analyzed using immunohistochemical staining from HumanProteinAtlas database. *P<0.05. Errors bars indicate respective standard deviations.

Next, we determined the expression of PIEZO2 in breast cancer cell lines and clinical samples compared with normal breast cell line and matched non-cancerous samples. Figure 2A showed that PIEZO2 expression in four breast cancer cell lines (MCF-7, Bcap37, MDA-MB-468 and MDA-MB-231) was significantly lower than that in normal breast cell line (HBL-100). Moreover, we found lower expression of PIEZO2 in high malignant cells (MDA-MB-468 and MDA-MB-231) compared with low malignant cell (MCF-7). Then, we compared the expression of PIEZO2 in breast cancer tissues with matched adjacent normal breast tissues and suggested that cancer tissues showed a significantly decreased PIEZO2 expression (Figure 2B). To further confirm under-expression of PIEZO2 in breast cancer, corresponding breast cancer expression data from TCGA were analyzed using UALCAN database (Figure 2C). Intriguingly, a similar result was observed. Furthermore, the protein level of PIEZO2 was also decreased in breast cancer tissues compared with normal breast tissues (Figure 2D).

**Figure 3:**
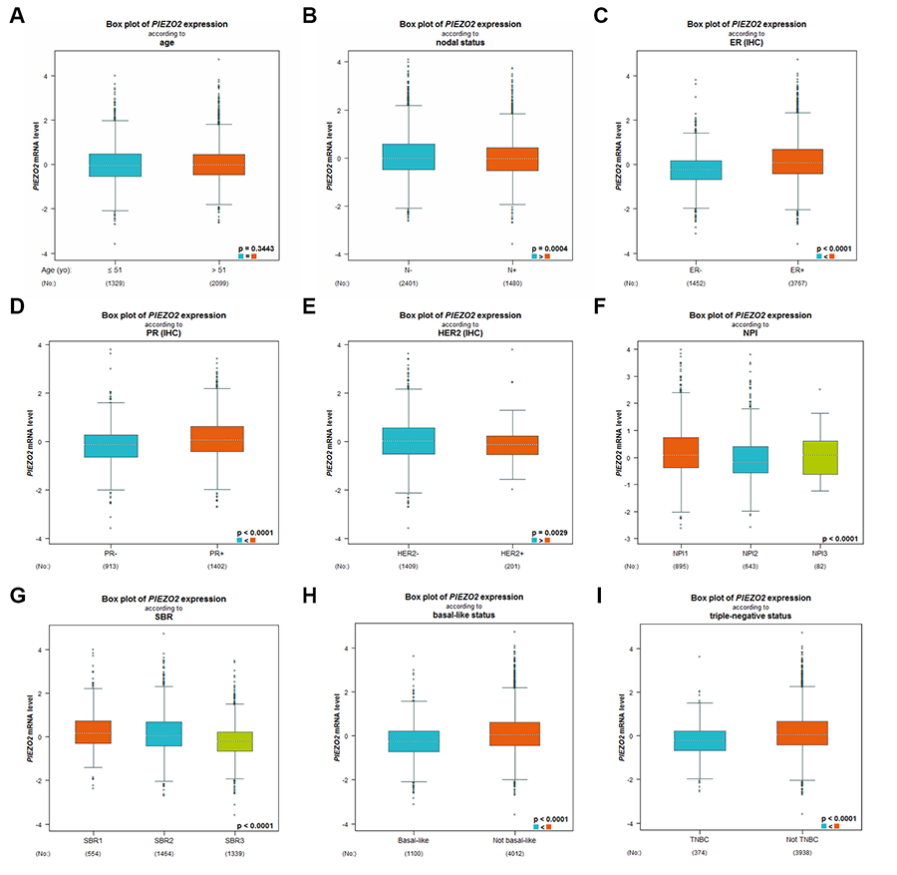
Expression differences of PIEZO2 in breast cancer patients based on different clinicopathological features, including age (**A**), nodal status (**B**), ER status (**C**), PR status (**D**), HER2 status (**E**), NPI score (**F**), SBR grade (**G**), basal-like status (**H**) and triple-negative status (**I**).

Subsequently, we further studied the expression differences of PIEZO2 based on different clinicopathological parameters in breast cancer using bc-GenExMiner database. As shown in Figure 3A, no significant difference between the <51 years and >51 years was observed. Figure 3 also demonstrated that PIEZO2 expression was markedly upregulated in breast cancer with nodal negative (Figure 3B), ER positive (Figure 3C), PR positive (Figure 3D) and HER2 negative (Figure 3E) status. PIEZO2 expression was inversely correlated with NPI score and SBR grade as presented in Figure 3F and Figure 3G, respectively. Additionally, we also discovered that expression of PIEZO2 was significantly decreased in basal-like (Figure 3H) and triple negative (Figure 3I) breast cancer patients compared with not basal-like and not triple negative breast cancer, respectively. We further investigated the relationship between PIEZO2 expression and clinicopathological characteristics using TCGA breast cancer data. Chi-square test revealed that PIEZO2 expression was significantly associated with ER status (P < 0.001), PR status (P < 0.001), HER2 status (P < 0.001), T stage (P < 0.001), N stage (P = 0.0221) and pathologic stage (P = 0.0010) (Table 1). All these findings suggest that PIEZO2 expression in breast cancer is significantly declined and negatively correlates with progression of breast cancer.

**Table 1:** Correlation between PIEZO2 expression and clinicopathological characteristics in TCGA breast cancer.

Features		Breast cancer		
		PIEZ02		
		low/high expression case (n)		^a^P value
Age at diagnosis	Cases			
<=51	367	283/84		0.6101
>51	729	572/157		
Estrogen receptor status				
Positive	806	596/210		**< 0.0001**
Negative	240	224/16		
NA	50	35/15		
Progesterone receptor status				
Positive	698	513/185		**< 0.0001**
Negative	345	304/41		
NA	53	38/15		
Her2 receptor status				
Positive	161	147/14		**< 0.0001**
Negative	564	425/139		
NA	371	283/88		
T stage				
T1	280	193/87		**< 0.0001**
T2/T3/T4	813	660/153		
TX	3	2/1		
N stage				
N0/N1	880	675/205		**0.0221**
N2/N3	196	165/31		
NX	20	15/5		
M stage				
M0	910	711/199		0.8316
M1	21	16/5		
MX	165	128/37		
Pathologic stage				
I	182	125/57		0.0010
II/III/IV	891	711/180		
NA	23	19/4		

^a^For analysis of relationship between PIEZO2 levels and various clinicopathological features, Pearson’s Chi-Square test was employed. If the expected count of variable was less than 5 and more than 1, Yates’ continuity corrected Chi-Square test was used. When the expected count of variable was less than 1, Fisher’s exact test was utilized.

The significant P value is marked with Bold type. NA=Not Applicable.

### Breast cancer patients with lower expression of PIEZO2 have poorer prognosis

**Figure 4:**
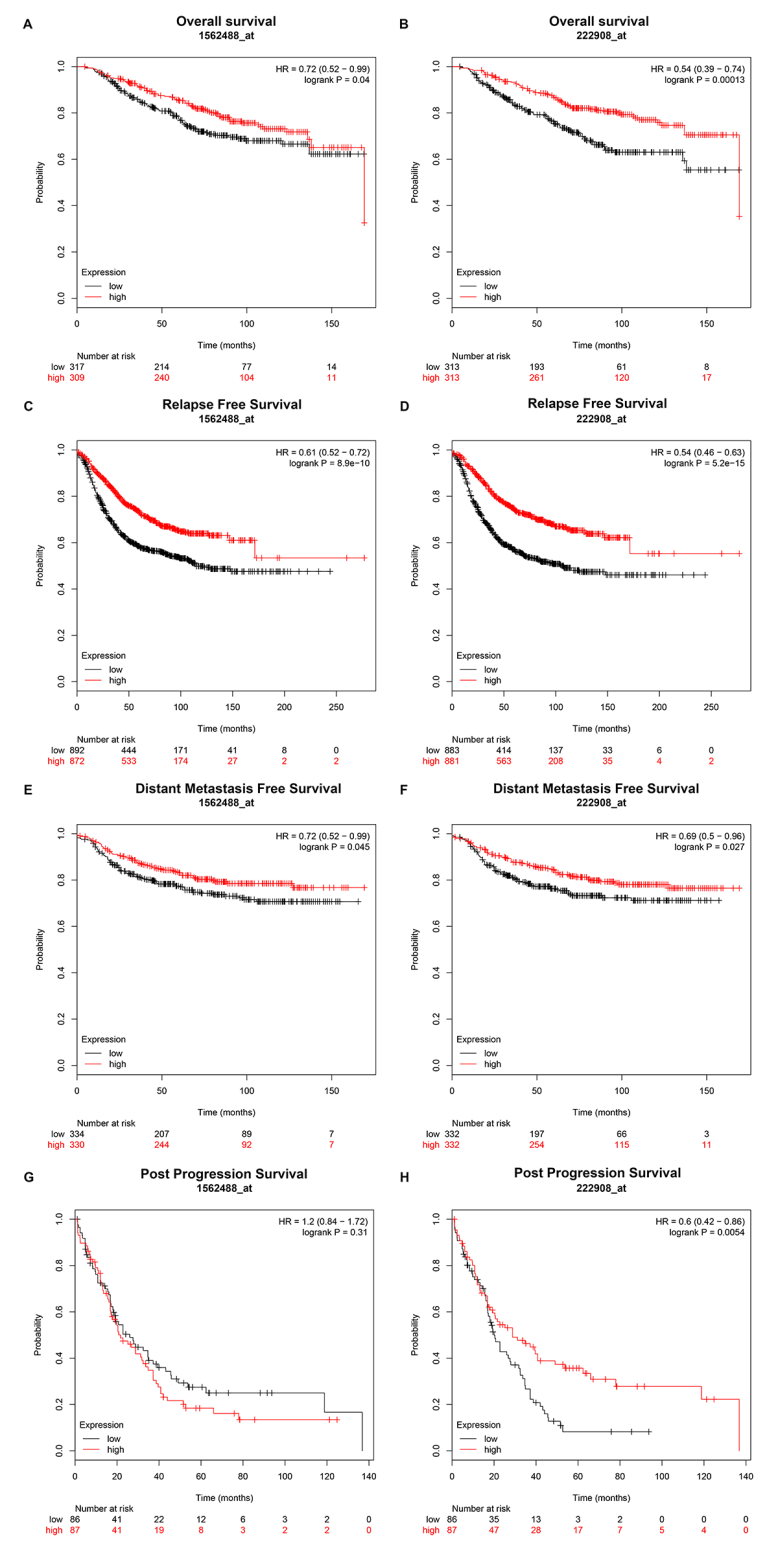
**Prognostic value of PIEZO2 (Affymetrix ID are valid: 1562488_at and 222908_at) in breast cancer patients.** (**A-B**) Overall survival curve of PIEZO2 for breast cancer patients; (**C-D**) relapse free survival curve of PIEZO2 for breast cancer patients; (**E-F**) distant metastases free survival curve of PIEZO2 for breast cancer patients; (**G-H**) post progression survival curve of PIEZO2 for breast cancer patients.

Then, we explored the prognostic values of PIEZO2 in breast cancer. When we entered PIEZO2 in Kaplan Meier-Plotter database, two probes (1562488_at and 222908_at) were found. As shown in Figure 4, breast cancer patients with high expression of PIEZO2 had a significantly favorable prognosis, including overall survival, relapse free survival and distant metastasis free survival in both two probes. Regarding to post progression survival, high expression of PIEZO2 was also found to be significantly linked to a favorable prognosis in the probe 222908_at. In the probe 1562488_at, however, no statistical significance of PIEZO2 for predicting post progression survival was observed between the PIEZO2 high expression group and PIEZO2 low expression group. Furthermore, we also assessed the relationship between PIEZO2 expression and prognosis in breast cancer using PrognoScan database (Table 2). As listed in Table 2, PIEZO2 expression level was positively correlated with distant metastasis free survival, relapse free survival, disease specific survival, disease free survival and overall survival in patients with breast cancer.

**Table 2:** Relationship between PIEZO2 expression and prognosis in breast cancer patients (PrognoScan database).

Dataset	Endpoint	Patient number	Probe ID	Cox P-value	HR(95% CI)
GSE11121	Distant Metastasis Free Survival	200	219602_s_at	0.002372	0.50(0.32-0.78)
GSE1456-GPL96	Relapse Free Survival	159	219602_s_at	0.004637	0.57(0.38-0.84)
GSE1456-GPL97	Relapse Free Survival	159	222908_at	0.012288	0.67(0.49-0.92)
GSE1456-GPL96	Disease Specific Survival	159	219602_at	0.017194	0.57(0.36-0.91)
GSE12276	Relapse Free Survival	204	1565775_at	0.027079	0.84(0.73-0.98)
GSE4922-GPL97	Disease Free Survival	249	222908_at	0.035685	0.82(-.68-0.99)
GSE1456-GPL97	Disease Specific Survival	159	222908_at	0.039966	0.68(0.47-0.98)
GSE1456-GPL96	Overall Survival	159	219602_s_at	0.044087	0.66(0.44-0.99)
GSE12276	Relapse Free Survival	204	219602_s_at	0.044552	0.83(0.69-1.00)

**Figure 5:**
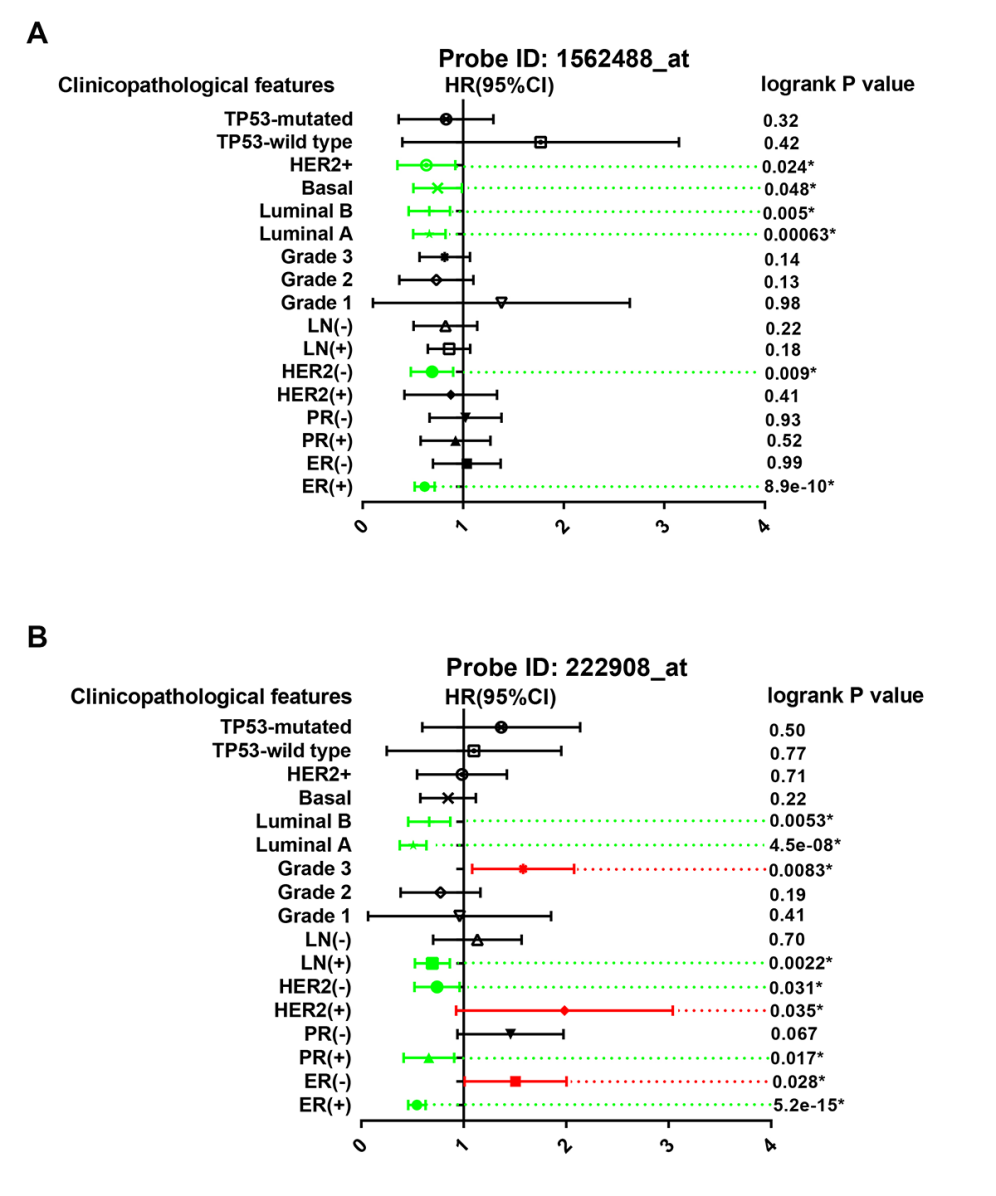
**Prognostic values of PIEZO2 in breast cancer patients based on different clinicopathological features.** Green bars indicate a favorable prognosis; red bars indicate an unfavorable prognosis; black bars represent no statistical significance.

The prognostic roles of PIEZO2 in breast cancer patients according to various clinicopathological features were also determined by Kaplan Meier-Plotter database. The data were presented in Figure 5 which demonstrated that PIEZO2 overexpression was significantly associated with better prognosis in ER-positive, HER2-negative, luminal A and luminal B breast cancer in the two probes. Furthermore, we also found that high expression of PIEZO2 in basal-like breast cancer patients indicated a better prognosis in the probe 1562488_at. In the probe 222908_at, a positive correlation of PIEZO2 expression and survival in PR-positive and lymph node-positive breast cancer patients but a negative association between PIEZO2 expression and survival in ER-negative, HER2-positive and pathological grade 3 breast cancer patients were observed.

### Identification of miRNAs that potentially regulate PIEZO2

**Figure 6:**
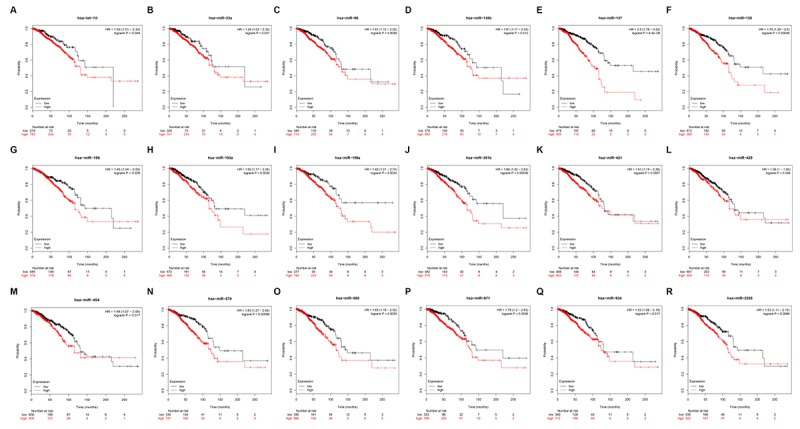
**Prognostic values of 18 potential miRNAs in breast cancer analyzed by Kaplan Meier-plotter database.**

To identify PIEZO2 that can be modulated by miRNAs, starBase database was utilized to predict upstream miRNAs of PIEZO2. In total, 182 miRNAs could potentially regulate PIEZO2 as shown in Table S1. It has been widely acknowledged that there exists an inverse association between miRNA and mRNA expression [12–15]. Therefore, we further assessed expression correlations between each predicted miRNA and PIEZO2 using TCGA breast cancer data. We observed that 42 miRNAs expression were statistically inversely associated with PIEZO2 expression (Table 3). Next, the prognostic values of the 42 miRNAs in breast cancer were determined by Kaplan Meier-Plotter database. The results demonstrated that breast cancer patients with high expression of 18 miRNAs (let-7d-5p, miR-33a-5p, miR-98-5p, miR-130b-3p, miR-137, miR-138-5p, miR-186-5p, miR-193a-3p, miR-196a-5p, miR-301a-3p, miR-421, miR-429, miR-454-3p, miR-579-3p, miR-580-3p, miR-671-5p, miR-934 and miR-2355-5p) indicated a poor prognosis (Figure 6). Moreover, we also detected the 18 miRNAs expression in breast cancer using OncomiR database. As listed in Table 4, only 11 miRNAs (let-7d-5p, miR-33a-5p, miR-98-5p, miR-130b-3p, miR-137, miR-193a-3p, miR-196a-5p, miR-301a-3p, miR-421, miR-454-3p and miR-671-5p) were significantly upregulated in breast cancer when compared with normal controls. Subsequently, by text mining for the 11 miRNAs, we found that 5 miRNAs (miR-130b-3p, miR-196a-5p, miR-301a-3p, miR-421 and miR-454-3p) were reported to function as oncogenes in breast cancer (Table 5). In order to further validate the inverse regulation of the five miRNAs in PIEZO2, we determined the mRNA and protein expression levels of PIEZO2 after knockdown of the five miRNAs. As shown in Figure S1, PIEZO2 was significantly upregulated in miRNAs-knockdown groups compared with negative control group. Taken together, the 5 miRNAs, with the features of upregulation in breast cancer, indicating poor prognosis, possessing negative associations with PIEZO2 expression and being reported to act as oncogenes, were the most potential upstream miRNAs that could inversely regulate PIEZO2 in breast cancer.

**Table 3:** The correlation between predicted miRNA and PIEZO2.

Predicted miRNA	R	P-value
Let-7d-5p	-0.197	5.48e-11
Let-7g-5p	-0.105	5.52e-04
Let-7i-5p	-0.133	1.07e-05
miR-7-5p	-0.198	5.17e-11
miR-15a-5p	-0.133	1.04e-05
miR-15b-5p	-0.125	3.51e-05
miR-25-3p	-0.184	9.23e-10
miR-27a-3p	-0.175	6.43e-09
miR-28-5p	-0.123	4.85e-05
miR-32-5p	-0.206	7.07e-12
miR-33a-5p	-0.196	8.19e-11
miR-92a-3p	-0.149	8.40e-07
miR-98-5p	-0.248	9.93e-17
miR-130b-3p	-0.198	5.25e-11
miR-137	-0.136	7.03e-06
miR-138-5p	-0.150	7.52e-07
miR-142-5p	-0.206	7.61e-12
miR-146b-5p	-0.252	3.82e-17
miR-186-5p	-0.180	2.36e-09
miR-193a-3p	-0.150	8.09e-04
miR-196a-5p	-0.129	2.05e-05
miR-197-3p	-0.178	3.31e-09
miR-200c-3p	-0.124	3.93e-05
miR-224-5p	-0.245	2.51e-16
miR-301a-3p	-0.219	3.00e-13
miR-301b-3p	-0.207	5.77e-12
miR-330-5p	-0.106	4.59e-04
miR-345-5p	-0.145	1.54e-06
miR-362-5p	-0.257	7.35e-18
miR-421	-0.224	3.85e-14
miR-429	-0.147	1.21e-16
miR-452-5p	-0.128	4.16e-03
miR-454-3p	-0.149	7.55e-07
miR-455-3p	-0.147	1.12e-06
miR-577	-0.232	9.38e-15
miR-579-3p	-0.101	8.67e-14
miR-580-3p	-0.148	1.05e-06
miR-589-5p	-0.214	1.02e-12
miR-671-5p	-0.197	6.54e-11
miR-708-5p	-0.115	1.04e-02
miR-934	-0.269	1.83e-19
miR-2355-5p	-0.159	1.50e-07

**Table 4:** The expression of potential miRNAs in breast cancer (OncomiR database).

MiRNA name	T-Test P-value	T-Test FDR	Tumor log2 mean expression	Normal log2 mean expression
let-7d-5p	3.49e-08	1.31e-07	7.75	7.20
miR-33a-5p	1.63e-14	1.13e-13	3.60	1.91
miR-98-5p	2.62e-14	1.79e-13	5.80	5.12
miR-130b-3p	1.04e-21	1.56e-20	3.36	1.82
miR-137	2.03e-02	3.59e-02	0.14	0.00
miR-193a-3p	7.57e-04	1.76e-03	4.05	3.63
miR-196a-5p	1.22e-18	1.35e-17	8.49	6.05
miR-301a-3p	2.42e-16	2.08e-15	3.16	1.54
miR-421	6.17e-03	1.23e-02	0.69	0.42
miR-454-3p	1.08e-19	1.31e-18	3.20	1.61
miR-671-5p	1.94e-24	4.79e-23	2.00	0.53

**Table 5:** Text mining the roles of potential miRNAs in breast cancer.

PubMed ID	miRNAs	Direct targets	Function	Sum effect	Refs
28165066	miR-130b-3p	PTEN	mediate drug resistance and proliferation	oncogenic	[41]
29685157	miR-196a-3p	SPRED1	promote tumor growth and metastasis	oncogenic	[42]
24315818	miR-301a-3p	PTEN	promote tumor metastasis	oncogenic	[43]
29763890	miR-301a-3p	ESR1	Suppress estrogen signaling	oncogenic	[44]
29396508	miR-301a-3p	-	Indicate a poor prognosis	oncogenic	[45]
25311065	miR-301a-3p	-	Indicate a poor prognosis	oncogenic	[46]
30365117	miR-421	PDCD4	promote proliferation	oncogenic	[47]
29322788	miR-421	Caspase-10	promote tumor progression	oncogenic	[48]
28795052	miR-454-3p	AKT	promote proliferation, migration, invasion and suppress apoptosis	oncogenic	[49]
27588500	miR-454-3p	-	Indicate a poor prognosis	oncogenic	[50]

### GO functional annotation and pathway enrichment analysis of co-expressed genes of PIEZO2

**Figure 7:**
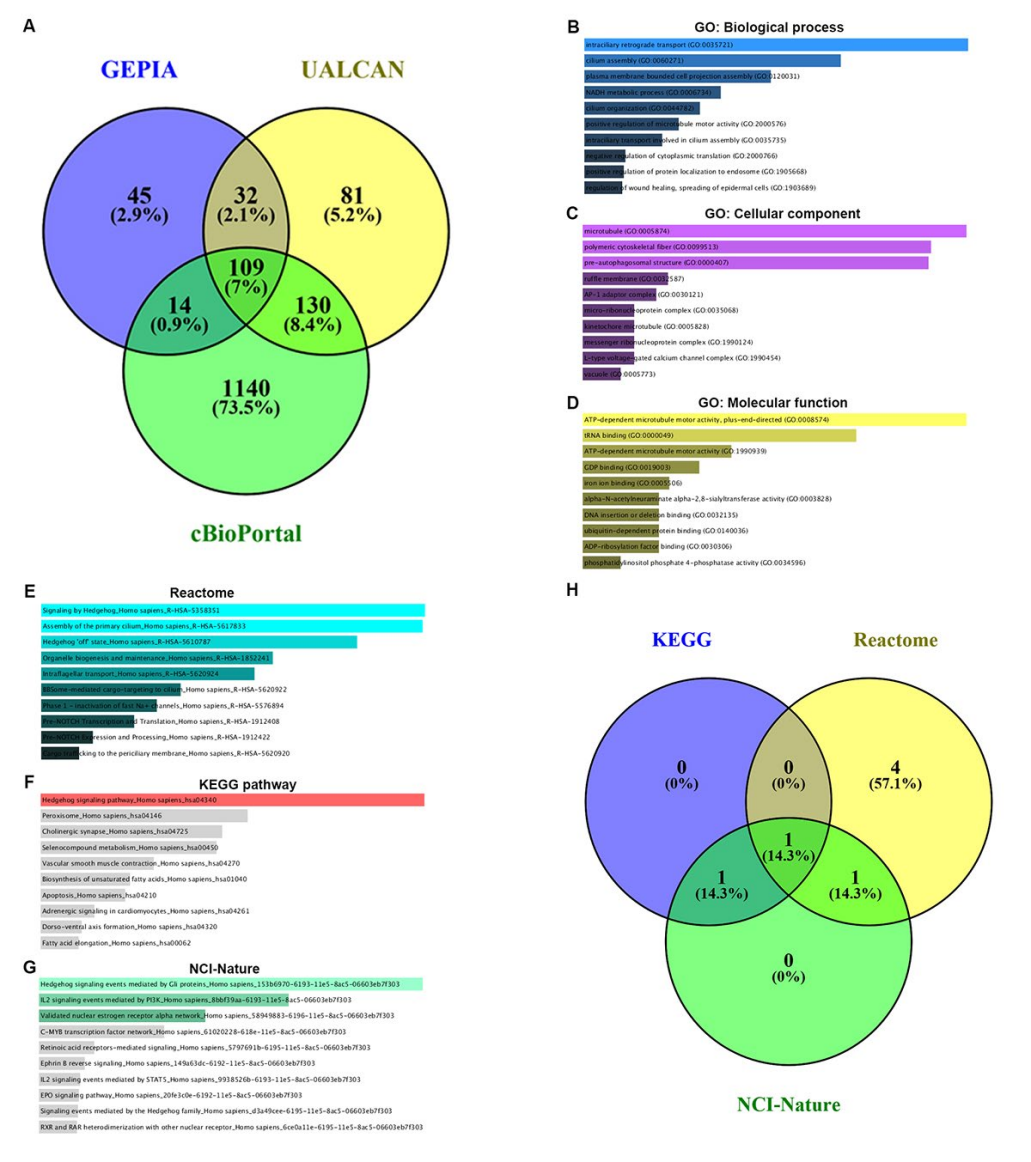
**GO functional annotation and pathway enrichment analysis for the co-expressed genes of PIEZO2.** (**A**) The Veen diagram of PIEZO2’s co-expressed genes from GEPIA, UALCAN and cBioPortal databases; (**B-D**) GO functional annotation (biological process, cellular component and molecular function) for the 109 co-expressed genes of PIEZO2; (**E-G**) pathway (Reactome, KEGG and NCI-Nature) enrichment analysis for the 109 co-expressed genes of PIEZO2; (**H**) the Veen diagram of the genes enriched in Hedgehog signaling pathway from Reactome, KEGG and NCI-Nature databases.

Co-expression of PIEZO2 was analyzed using three databases, namely GEPIA, UALCAN and cBioPortal. As shown in Figure 7A, 109 co-expressed genes of PIEZO2 were commonly appeared in all the three databases. To better understand these genes, GO functional annotation and pathway enrichment analysis were conducted using Enrichr database. Three GO categories, containing biological process, cellular component and molecular function, were included in the functional annotation. For pathway enrichment, Reactome’s cell signaling pathway, KEGG’s cell signaling pathway and cell signaling pathway from NCI-Nature were analyzed.

The top ten enriched GO terms were shown in Figure 7B-D, including intraciliary retrograde transport, cilium assembly and plasma membrane bounded cell projection assembly in the biological process category, microtubule, polymetric cytoskeletal fiber and pre-autophagosomal structure in the cellular component category and ATP-dependent microtubule motor activity plus-end-directed, tRNA binding and ATP-dependent microtubule motor activity in the molecular function category. The top ten enriched pathways in three pathway categories were presented in Figure 7E-G. Intriguingly, we found that Hedgehog signaling pathway was the most top enriched pathway in all the three pathway databases. The corresponding gene counts of Hedgehog signaling pathway in Reactome, KEGG and NCI-Nature pathways were displayed in Figure 7H. A total of seven genes (IFT88, INTU, WDR19, WDR35, GLI3, CDON and CSNK1G3) were enriched in Hedgehog signaling pathway.

### Decreased expression of PIEZO2 correlates with dysregulation of Hedgehog signaling pathway

**Figure 8:**
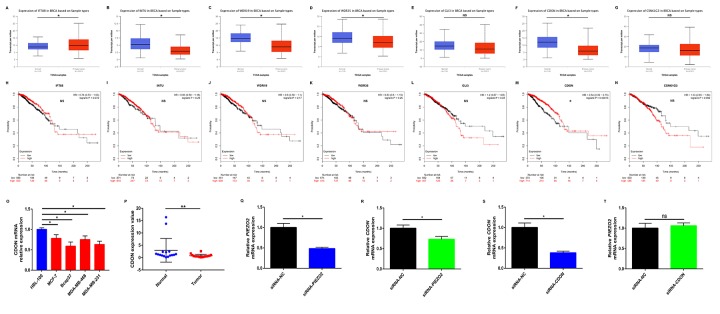
**Identification of potential downstream of PIEZO2 in breast cancer.** (**A-G**) Expression of IFT88, INTU, WDR19, WDR35, GLI3, CDON and CSNK1G3 in breast cancer analyzed using UALCAN; (**H-N**) prognostic roles of IFT88, INTU, WDR19, WDR35, GLI3, CDON and CSNK1G3 in breast cancer analyzed using Kaplan Meier-Plotter; (**O**) CDON expression in human breast cancer cell lines (MCF-7, Bcap37, MDA-MB-468 and MDA-MB-231) compared with that in normal breast cell line (HBL-100); (**P**) CDON expression in clinical breast cancer tissues compared with that in matched adjacent normal tissues (n=16); (**Q**) knockdown effect of siRNA-PIEZO2 in MCF-7 cell line; (**R**) expression change of CDON after silencing expression of PIEZO2 in MCF-7 cell line; (**S**) knockdown effect of siRNA-CDON in MCF-7 cell line; (**T**) expression change of PIEZO2 after silencing expression of CDON in MCF-7 cell line. *P<0.05; **P<0.01; “NS” represents no statistical significance.

To preliminarily explore the role of Hedgehog signaling pathway in PIEZO2-mediated progression of breast cancer, we first determined the expression of the seven genes enriched in Hedgehog signaling pathway in breast cancer using UALCAN database as shown in Figure 8A-G. Among the seven genes, an obvious upregulation of IFT88 (Figure 8A) in breast cancer was found whereas INTU (Figure 8B), WDR19 (Figure 8C), WDR35 (Figure 8D) and CDON (Figure 8F) expression levels in breast cancer samples were significantly lower than that in normal breast samples. GLI3 (Figure 8E) and CSNK1G3 (Figure 8G) showed no significant differences between cancer tissues and normal tissues. Subsequently, we further evaluated the prognostic values of the seven genes in breast cancer patients by Kaplan Meier-Plotter database (Figure 8H-N). The results showed that breast cancer patients with higher expression of CDON indicated a better prognosis. However, for the other six genes, no statistical significance was found. By combination of expression and prognostic roles of these genes, CDON was thought to closely correlate with PIEZO2 in breast cancer. The positive correlation of CDON expression with PIEZO2 expression in breast cancer was further determined by four databases, namely GEPIA, UALCAN, bc-GenExMiner and cBioPortal (Figure S2). Moreover, we also employed Oncomine database and Human Protein Atlas database to further testify the expression of CDON in breast cancer. As shown in Figure S3A-E, CDON mRNA expression was significantly lower in invasive breast carcinoma, invasive ductal breast carcinoma and invasive lobular breast carcinoma. Figure S3F demonstrated that expression level of CDON protein was also markedly decreased in breast cancer. Subsequently, CDON expression among groups of patients, based on various ER, PR, HER2, nodal, basal-like and triple negative status were determined using bc-GenExMiner. The analytic data were presented in Table 6. In breast cancer patients with positive ER, positive PR, negative HER2, negative nodal, negative basal-like and negative triple-negative status, expression of CDON was significantly upregulated when compared with corresponding counterparts. Furthermore, we also experimentally demonstrated the expression of CDON in breast cancer cell lines (Figure 8O) and clinical breast cancer samples (Figure 8P). The results also revealed that CDON expression was significantly downregulated in breast cancer cell lines and clinical cancer samples when compared with their counterparts. To preliminarily explore the upstream and downstream association of CDON and PIEZO2 in breast cancer, we detected the expression change of CDON or PIEZO2 after silencing expression of PIEZO2 or CDON using siRNA-PIEZO2 or siRNA-CDON, respectively. In this study, MCF-7 was chosen as the representative breast cancer cell line as its relatively high expression of PIEZO2 and CDON compared with other cell lines (Figure 2A and Figure 8O). The knockdown effects of siRNA-PIEZO2 and siRNA-CDON were presented in Figure 8Q and Figure 8S, respectively. Figure 8R showed a significant reduction of CDON expression after knockdown of PIEZO2. However, PIEZO2 expression was not statistically changed after downregulation of CDON (Figure 8T). All these findings indicate that PIEZO2 might be associated with Hedgehog signaling pathway by regulating CDON in breast cancer.

**Table 6:** The relationship between CDON and clinicopathological parameters of breast carcinoma.

Variables		CDON	
	Number	mRNA expression	P-value
ER			**<0.0001**
			
-	1525	-	
+	3923	Up	
PR			**<0.0001**
-	946	-	
+	1439	Up	
HER2			**<0.0001**
-	1409	Up	
+	201	-	
Nodal Statas			**0.0064**
+	1509	-	
-	2447	Up	
Basal-like Status			**<0.0001**
Not	4200	Up	
Basal-like	1144	-	
Triple-negative Status			**0.0005**
Not	3299	Up	
TNBC	293	-	

## DISCUSSION

PIEZO2, a mechanically activated ion channel, is believed to play important roles in the onset and progression of human cancers for a long time. Recently, several studies have reported that the dysregulation of PIEZO2 is associated with cancer proliferation, angiogenesis and resistance to anticancer treatments [9,11,16]. However, to date, the expression, prognostic value and underlying mechanisms of PIEZO2 in cancer remain largely unknown.

By analyzing expression profile of PIEZO2 using the HPA database, we found that breast cancer was the most suitable candidate for further investigation. Moreover, the role of PIEZO2 expression in the development and progression of breast cancer has not been identified. Subsequently, we confirmed that PIEZO2 was frequently downregulated in breast cancer cell lines and clinical samples relative to corresponding normal cell line and matched adjacent normal samples using qRT-PCR. UALCAN database analysis also revealed a low expression of PIEZO2 in breast cancer. Meanwhile, PIEZO2 expression was found to positively correlate with ER status and PR status but negatively correlate with HER2 status, NPI score, SBR grade, basal-like status and triple-negative status in breast cancer, indicating that high expression of PIEZO2 is closely linked to progression of breast cancer. Using Kaplan Meier-Plotter, we found a favorable prognosis of PIEZO2 expression in breast cancer, especially in ER-positive, HER2-negative, luminal A and luminal B breast cancer. We also investigated the prognostic role of PIEZO2 in breast cancer by PrognoScan, and the results demonstrated that breast cancer patients with low expression of PIEZO2 had a poor prognosis. Altogether, these findings suggest that low expression of PIEZO2 might be a promising prognostic biomarker in breast cancer.

**Figure 9:**
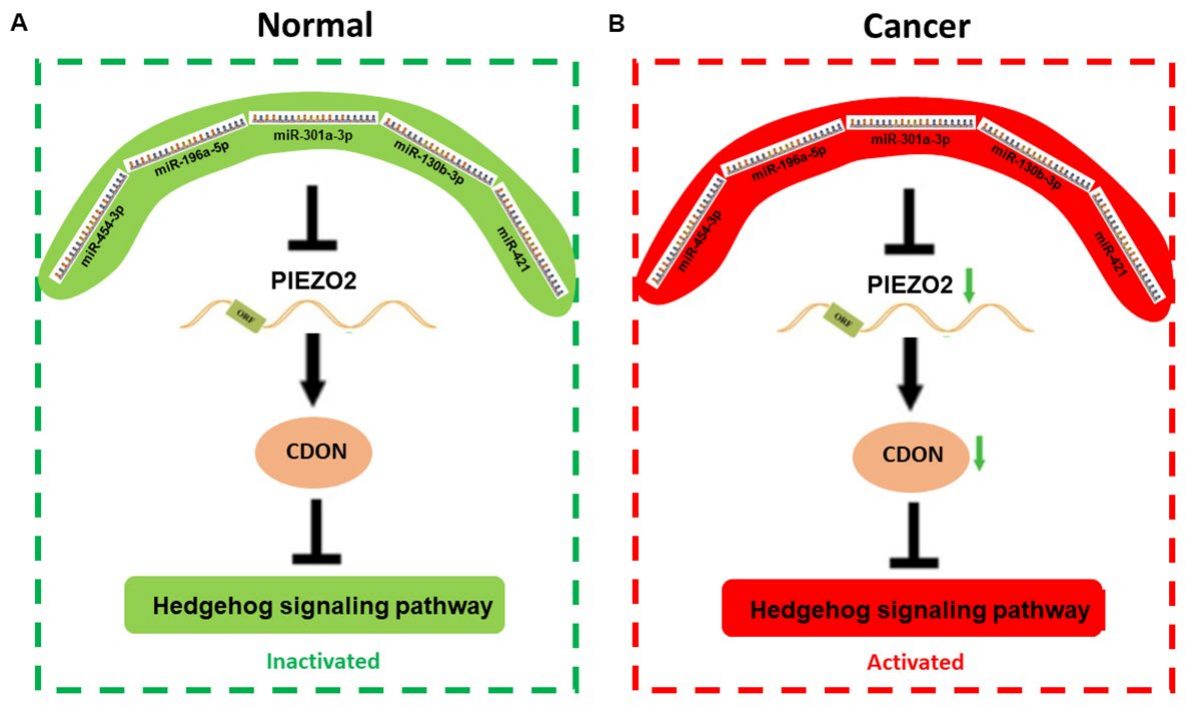
The mechanism graph of the regulatory network of PIEZO2 under different circumstance including normal (**A**) and cancer (**B**). In patients with breast cancer, miRNAs (miR-454-3p, miR-196a-5p, miR-301a-3p, miR-130b-3p and miR-421)-mediated downregulation of PIEZO2 can activate hedgehog signaling pathway by suppressing CDON.

Next, we explored the underlying mechanism how PIEZO2 exerted its roles in breast cancer (Figure 9). It is known to all that genes can be post-transcriptionally regulated by miRNAs. 182 upstream miRNAs were first predicted to potentially regulate PIEZO2. Among these miRNAs, we found that five miRNAs (miR-130b-3p, miR-196a-3p, miR-301a-3p, miR-421 and miR-454-3p) possessed the greatest potential in targeting PIEZO2 in breast cancer by combination of correlation analysis, prognosis analysis, expression analysis and text mining.

By co-expression analysis, 109 co-expressed genes were also identified. Pathway enrichment analysis showed that these co-expressed genes were significantly enriched in Hedgehog signaling pathway. Numerous studies have suggested that activation of Hedgehog signaling pathway is implicated in the development of a variety of human cancers, including breast cancer [17–20]. In total, seven genes (IFT88, INTU, WDR19, WDR35, GLI3, CDON and CSNK1G3) were enriched in Hedgehog signaling pathway. We further determined expression and prognostic roles of the seven genes in breast cancer using TCGA breast cancer data via analyzing UALCAN and Kaplan Meier-Plotter. Among these genes, only CDON was downregulated in breast cancer and the decreased expression of CDON indicated a poor prognosis. CDON, a receptor of Sonic Hedgehog, has been found to block tumor growth and progression by inducing apoptosis [21,22]. The downregulation of CDON expression in breast cancer was experimentally validated in our breast cancer cell lines and clinical breast cancer samples. Moreover, we also demonstrated that CDON expression was significantly decreased after knockdown of PIEZO2, preliminarily suggesting that CDON acts as a downstream of PIEZO2. All these results together provide plenitudinous evidence that PIEZO2, downregulated by five oncogenic miRNAs (miR-130b-3p, miR-196a-3p, miR-301a-3p, miR-421 and miR-454-3p), might promote survival and progression of breast cancer by decreasing expression of CDON.

In conclusion, the present study confirmed that PIEZO2 expression in breast cancer was decreased and the downregulated expression of PIEZO2 indicated a poor prognosis of patients with breast cancer. Furthermore, we also found that PIEZO2 expression was potentially targeted by five miRNAs and correlated with dysregulation of Hedgehog signaling pathway, especially the SHH co-receptor-CDON. Of course, these findings should be validated in large-scale comprehensive studies and multicenter clinical trials in the future.

## MATERIALS AND METHODS

### Human Protein Atlas database analysis

Expression of PIEZO2 mRNA and protein in different human normal and cancer tissues were determined using the Human Protein Atlas (HPA) database [23]. CDON protein expression level in breast cancer tissues and normal breast tissues was also analyzed using HPA database.

### UALCAN database analysis

UALCAN database, a user-friendly and interactive web resource, provides easy access to publicly available cancer transcriptome data from The Cancer Genome Atlas (TCGA) [24]. In this study, it was utilized to analyze gene expression and assess the correlation between two genes. Statistical analysis was calculated and displayed on the webpage and logrank P-value < 0.05 was considered as statistically significant.

### Breast cancer gene expression miner

Breast cancer gene expression miner (bc-GenExMiner) is an easy-to-use online platform for analyzing gene expression, prognosis and correlation in breast cancer [25,26]. Bc-GenExMiner was introduced to determine PIEZO2 and CDON expression in breast cancer based on different clinicopathological features. Moreover, correlation of PIEZO2 and CDON was also assessed by bc-GenExMiner. P-value < 0.05 was considered as statistically significant.

### Kaplan Meier-Plotter database analysis

Kaplan Meier-Plotter database is established using gene expression data and survival information of cancer patients downloaded from the Gene Expression Omnibus database [27]. The database was used to analyze associations between PIEZO2 expression and overall survival, relapse-free survival, distant metastases-free survival or post-progression survival in breast cancer. In this study, briefly, PIEZO2 was firstly entered into the database to obtain Kaplan Meier survival plots. PIEZO2 expression above or below the median classified these cases into a low expression group and a high expression group. These cohorts were then compared with a Kaplan-Meier survival plot, and hazard ratio (HR), 95% confidence interval (CI), and logrank P-value were determined and displayed on the webpage. In addition, the prognostic values of predicted miRNAs, IFT88, INTU, WDR19, WDR35, GLI3, CDON and CSNK1G3 in breast cancer were also evaluated using Kaplan Meier-Plotter database. A logrank P-value < 0.05 was considered as statistically significant.

### PrognoScan database analysis

The correlation between the expression of PIEZO2 and survival in breast cancer was also determined using the PrognoScan database which is a database for meta-analysis of the prognostic values of genes in human cancers, including bladder cancer, blood cancer, brain cancer, breast cancer, colorectal cancer, esophagus cancer, head and neck cancer, lung cancer, ovarian cancer, prostate cancer, renal cell carcinoma, skin cancer and soft tissue cancer [28,29]. A Cox P-value < 0.05 was considered as statistically significant.

### starBase database

The upstream miRNAs of PIEZO2 were predicted using starBase database, which is an open-source platform for studying the miRNA-ncRNA, miRNA-mRNA, ncRNA-RNA, RNA-RNA, RBP-ncRNA and RBP-mRNA interactions from CLIP-seq, degradome-seq and RNA-RNA interactome data [30,31]. starBase database was also employed to assess the inverse correlations of each miRNA expression and PIEZO2 expression. R < -0.1 and P-value < 0.05 were set as the thresholds for further identifying potential miRNAs that could target PIEZO2.

### OncomiR database analysis

OncomiR database is an online resource for exploring pan-cancer microRNA dysregulation in cancer [32]. miRNA expression in breast cancer was determined using OncomiR database. P-value < 0.05 was considered as statistically significant.

### GEPIA database analysis

GEPIA, a newly developed interactive web server for analyzing the RNA sequencing expression data of 9,736 tumors and 8,587 normal samples from the TCGA and the GTEx projects, was used to identify the co-expressed genes of PIEZO2 in breast cancer [33]. The similar genes listed on the webpage were directly downloaded.

### cBioPortal database analysis

cBioPortal is an online database for integrative analysis of complex cancer genomics and clinical profiles, which currently provides access to data from more than 48,668 tumor samples from 172 cancer studies in the TCGA pipeline [34,35]. It was also used to obtain the co-expressed genes of PIEZO2 in breast cancer. Only correlated genes with PCC > 0.3 were selected for subsequent investigation.

### Enrichr database analysis

Enrichr, a comprehensive gene set enrichment analysis web server, was employed to conduct Gene Ontology (GO) functional annotation and pathway enrichment analysis for these commonly appeared co-expressed genes of PIEZO2 [36,37]. The top ten enriched GO items and pathways were displayed on the webpage and directly downloaded.

### Oncomine analysis

Oncomine database is a web-based data mining platform for cancer research [38]. The expression of CDON in breast cancer was evaluated by Oncomine analysis of Cancer vs. Normal and meta-analysis. P-value < 0.0001 and fold change > 1.5 were set as the thresholds.

### Cell culture

All cell lines (HBL-100, MCF-7, Bcap37, MDA-MB-468 and MDA-MB-231) used in this study were purchased from the cell bank of Chinese Scientific Academy (Shanghai, China). HBL-100, MDA-MB-468 and MDA-MB-231 were cultured in Dulbecco’s modified Eagle’s medium (DMEM; Gibco, 12430047) supplemented with 10% fetal bovine serum (FBS; Biological Industries, 04-0101-1, Cromwell, CT, USA) and MCF-7 and Bcap37 were maintained in Roswell Park Memorial Institute (RPMI) 1640 medium (Gibco, 31800105, Life Technologies, Carlsbad) containing 10% FBS under a humidified atmosphere of 5% CO_2_ at 37°C.

### Patient and sample collection

16 clinical breast cancer tissues and matched adjacent normal tissues were collected from breast cancer patients who underwent surgery at the Zhejiang Cancer Hospital (Hangzhou, China) between 2016 and 2017. All procedures performed in this study involving human participants were conducted in accordance with the ethical standards of the Zhejiang Cancer Hospital and written informed consent from every participant was obtained.

### Cell transfection

Cell transfection was performed as we previously described [12,13,39]. miRNA inhibitors, siRNAs and their negative control oligonucleotides (NC) were purchased from RiboBio Co. Ltd (Guangzhou, China). 2 x 10^5^ of MCF-7 cells were seeded onto six-well plates, and cultured for 12 hours under a humidified atmosphere of 5% CO_2_ at 37°C. Subsequently, these cells were transfected with 50 nM of these oligonucleotides using Liopfectamine 3000 reagent (Invitrogen, Shanghai, China) according to the manufacturer’s instructions. The sequences of siRNA used in this work were listed in Table S2.

### RNA isolation and quantitative real-time polymerase chain reaction

RNA isolation and quantitative real-time polymerase chain reaction (qRT-PCR) were performed as described previously [12]. RNAiso plus Reagent (TaKaRa biotechnology, 9109, Kusatsu, Japan) was utilized to extract total RNA from cell lines and clinical samples. Then, total RNA was reversely transcribed into complementary DNA (cDNA) by the PrimeScript^TM^ RT Reagent Kit (TaKaRa biotechnology, RR037A). Subsequently, the cDNA was used for real-time polymerase chain reaction (RT-PCR) analysis with gene-specific primers. Real-time PCR was performed in a Roche LightCycle480 II Real-Time PCR Detection System through SYBR Premix Ex Taq (TaKaRa biotechnology, RR420A). Glyceralddehyde-2-phosphate dehydrogenase (GAPDH) and U6 was used as the internal control for genes and miRNAs, respectively. The primers used in this study were listed in Table S2. The expression of PIEZO2 or miRNAs was normalized to GAPDH or U6, and calculated using the comparative threshold method (2^−ΔΔCT^).

### Western blot

Western blot assay was performed to determine PIEZO2 protein level as we previously described [40]. β-Actin was used for normalization of protein loading. Experiments were repeated at least three times.

### Statistical analysis

All experiments were performed in triplicates. GraphPad prism 7 software (GraphPad Software, Inc., LaToIIa, CA, USA) was used to analyze expression data for statistical significance. The results were shown as mean ± SD. Differences between two groups were determined using Student’s *t*-test. The Chi-Square test was applied to the examination of relationship between PIEZO2 levels and clinicopathological features. A P-value < 0.05 was considered as statistically significant.

## SUPPLEMENTARY MATERIAL

Supplementary Table S1

Supplementary File
