# Supplementary material for: Five miRNAs-mediated PIEZO2 downregulation, accompanied with activation of Hedgehog signaling pathway, predicts poor prognosis of breast cancer
Source: Aging (Albany NY). 2019 May 6;11(9):2628–52. doi: 10.18632/aging.101934 (PMC6535055; doi:10.18632/aging.101934)
Supplement: Supplementary Table S1 [file aging-11-101934-s001.docx]

**Table S1. Potential miRNAs that regulate PIEZO2 (Predicted by starBase database).**

| Predicted miRNAs |
| --- |
| hsa-let-7a-5p |
| hsa-let-7b-5p |
| hsa-let-7c-5p |
| hsa-let-7d-5p |
| hsa-let-7e-5p |
| hsa-let-7f-5p |
| hsa-let-7g-5p |
| hsa-let-7i-5p |
| hsa-miR-103a-3p |
| hsa-miR-107 |
| hsa-miR-122-5p |
| hsa-miR-124-3p |
| hsa-miR-1247-5p |
| hsa-miR-1252-5p |
| hsa-miR-125a-5p |
| hsa-miR-125b-5p |
| hsa-miR-1271-5p |
| hsa-miR-1286 |
| hsa-miR-129-1-3p |
| hsa-miR-129-2-3p |
| hsa-miR-129-5p |
| hsa-miR-130a-3p |
| hsa-miR-130a-5p |
| hsa-miR-130b-3p |
| hsa-miR-1343-3p |
| hsa-miR-137 |
| hsa-miR-138-5p |
| hsa-miR-142-5p |
| hsa-miR-145-5p |
| hsa-miR-146a-5p |
| hsa-miR-146b-5p |
| hsa-miR-149-5p |
| hsa-miR-153-3p |
| hsa-miR-15a-5p |
| hsa-miR-15b-5p |
| hsa-miR-16-5p |
| hsa-miR-182-5p |
| hsa-miR-184 |
| hsa-miR-186-5p |
| hsa-miR-1913 |
| hsa-miR-193a-3p |
| hsa-miR-193b-3p |
| hsa-miR-195-5p |
| hsa-miR-196a-5p |
| hsa-miR-196b-5p |
| hsa-miR-197-3p |
| hsa-miR-199a-5p |
| hsa-miR-199b-5p |
| hsa-miR-200b-3p |
| hsa-miR-200c-3p |
| hsa-miR-2115-3p |
| hsa-miR-212-5p |
| hsa-miR-214-3p |
| hsa-miR-217 |
| hsa-miR-218-5p |
| hsa-miR-221-3p |
| hsa-miR-222-3p |
| hsa-miR-224-5p |
| hsa-miR-2355-5p |
| hsa-miR-25-3p |
| hsa-miR-2682-5p |
| hsa-miR-27a-3p |
| hsa-miR-27b-3p |
| hsa-miR-28-3p |
| hsa-miR-28-5p |
| hsa-miR-296-5p |
| hsa-miR-301a-3p |
| hsa-miR-301b-3p |
| hsa-miR-302a-3p |
| hsa-miR-302b-3p |
| hsa-miR-302c-3p |
| hsa-miR-302d-3p |
| hsa-miR-302e |
| hsa-miR-3064-5p |
| hsa-miR-30a-5p |
| hsa-miR-30b-5p |
| hsa-miR-30c-5p |
| hsa-miR-30d-5p |
| hsa-miR-30e-5p |
| hsa-miR-3139 |
| hsa-miR-3140-3p |
| hsa-miR-3173-5p |
| hsa-miR-3179 |
| hsa-miR-324-3p |
| hsa-miR-32-5p |
| hsa-miR-326 |
| hsa-miR-330-5p |
| hsa-miR-331-3p |
| hsa-miR-337-3p |
| hsa-miR-339-3p |
| hsa-miR-33a-5p |
| hsa-miR-33b-5p |
| hsa-miR-345-3p |
| hsa-miR-345-5p |
| hsa-miR-346 |
| hsa-miR-34a-5p |
| hsa-miR-34b-5p |
| hsa-miR-34c-5p |
| hsa-miR-3605-5p |
| hsa-miR-361-5p |
| hsa-miR-3619-5p |
| hsa-miR-362-5p |
| hsa-miR-363-3p |
| hsa-miR-367-3p |
| hsa-miR-369-3p |
| hsa-miR-372-3p |
| hsa-miR-373-3p |
| hsa-miR-379-3p |
| hsa-miR-382-3p |
| hsa-miR-383-5p |
| hsa-miR-3909 |
| hsa-miR-411-3p |
| hsa-miR-421 |
| hsa-miR-424-5p |
| hsa-miR-429 |
| hsa-miR-4319 |
| hsa-miR-4424 |
| hsa-miR-4458 |
| hsa-miR-449a |
| hsa-miR-449b-5p |
| hsa-miR-449c-5p |
| hsa-miR-4500 |
| hsa-miR-450b-5p |
| hsa-miR-452-5p |
| hsa-miR-454-3p |
| hsa-miR-455-3p |
| hsa-miR-4676-3p |
| hsa-miR-4712-5p |
| hsa-miR-485-5p |
| hsa-miR-491-5p |
| hsa-miR-493-5p |
| hsa-miR-494-3p |
| hsa-miR-495-3p |
| hsa-miR-496 |
| hsa-miR-497-5p |
| hsa-miR-503-5p |
| hsa-miR-506-3p |
| hsa-miR-513a-5p |
| hsa-miR-513b-5p |
| hsa-miR-515-5p |
| hsa-miR-5195-3p |
| hsa-miR-519a-3p |
| hsa-miR-519b-3p |
| hsa-miR-519c-3p |
| hsa-miR-520a-3p |
| hsa-miR-520b |
| hsa-miR-520c-3p |
| hsa-miR-520d-3p |
| hsa-miR-520d-5p |
| hsa-miR-520e |
| hsa-miR-522-3p |
| hsa-miR-524-5p |
| hsa-miR-543 |
| hsa-miR-552-3p |
| hsa-miR-556-3p |
| hsa-miR-5581-3p |
| hsa-miR-5586-5p |
| hsa-miR-577 |
| hsa-miR-579-3p |
| hsa-miR-580-3p |
| hsa-miR-589-5p |
| hsa-miR-616-3p |
| hsa-miR-642a-5p |
| hsa-miR-642b-5p |
| hsa-miR-653-5p |
| hsa-miR-655-3p |
| hsa-miR-656-3p |
| hsa-miR-664b-3p |
| hsa-miR-665 |
| hsa-miR-668-3p |
| hsa-miR-671-5p |
| hsa-miR-708-5p |
| hsa-miR-7-5p |
| hsa-miR-761 |
| hsa-miR-770-5p |
| hsa-miR-873-5p |
| hsa-miR-92a-3p |
| hsa-miR-92b-3p |
| hsa-miR-934 |
| hsa-miR-942-5p |
| hsa-miR-96-5p |
| hsa-miR-98-5p |
