# Supplementary material for: Five miRNAs-mediated PIEZO2 downregulation, accompanied with activation of Hedgehog signaling pathway, predicts poor prognosis of breast cancer
Source: Aging (Albany NY). 2019 May 6;11(9):2628–52. doi: 10.18632/aging.101934 (PMC6535055; doi:10.18632/aging.101934)
Supplement: Supplementary File [file aging-11-101934-s002.pdf]

SUPPLEMENTARY MATERIAL

Please browse the links in Full Text version of this manuscript to see Supplementary Table S1.

Table S1. Potential miRNAs that regulate PIEZO2 (Predicted by starBase database).

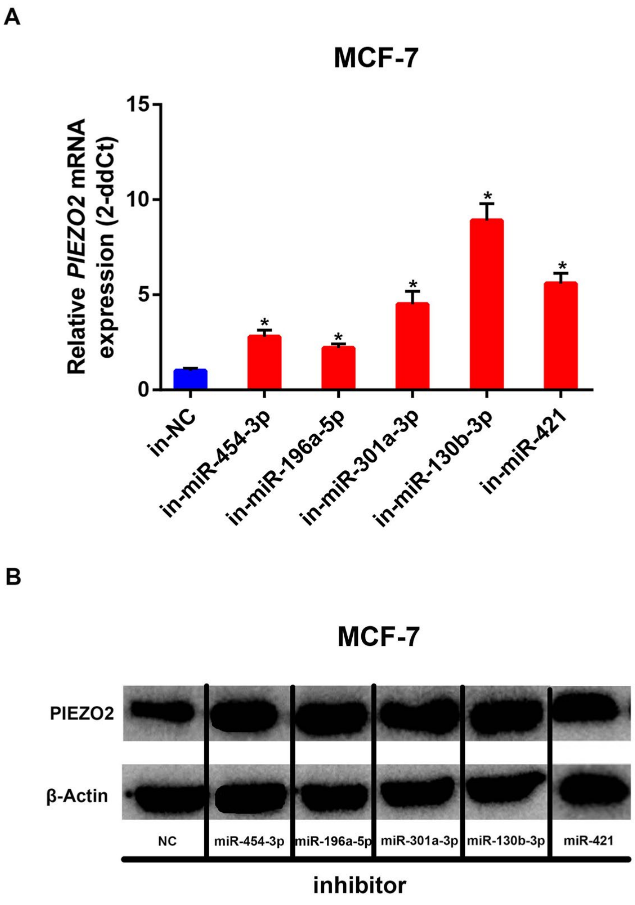

Figure S1. PIEZO2 expression changes after knockdown of the five potential upstream miRNAs. (**A**) The mRNA expression levels of PIEZO2 in MCF-7 treated with inhibitors for miR-454-3p, miR-196a-5p, miR-301a-3p, miR-130b-3p, miR-421 and negative control; (**B**) the protein expression levels of PIEZO2 in MCF-7 treated with inhibitors for miR-454-3p, miR-196a-5p, miR-301a-3p, miR-130b-3p, miR-421 and negative control. *P<0.05. Errors bars indicate respective standard deviations.

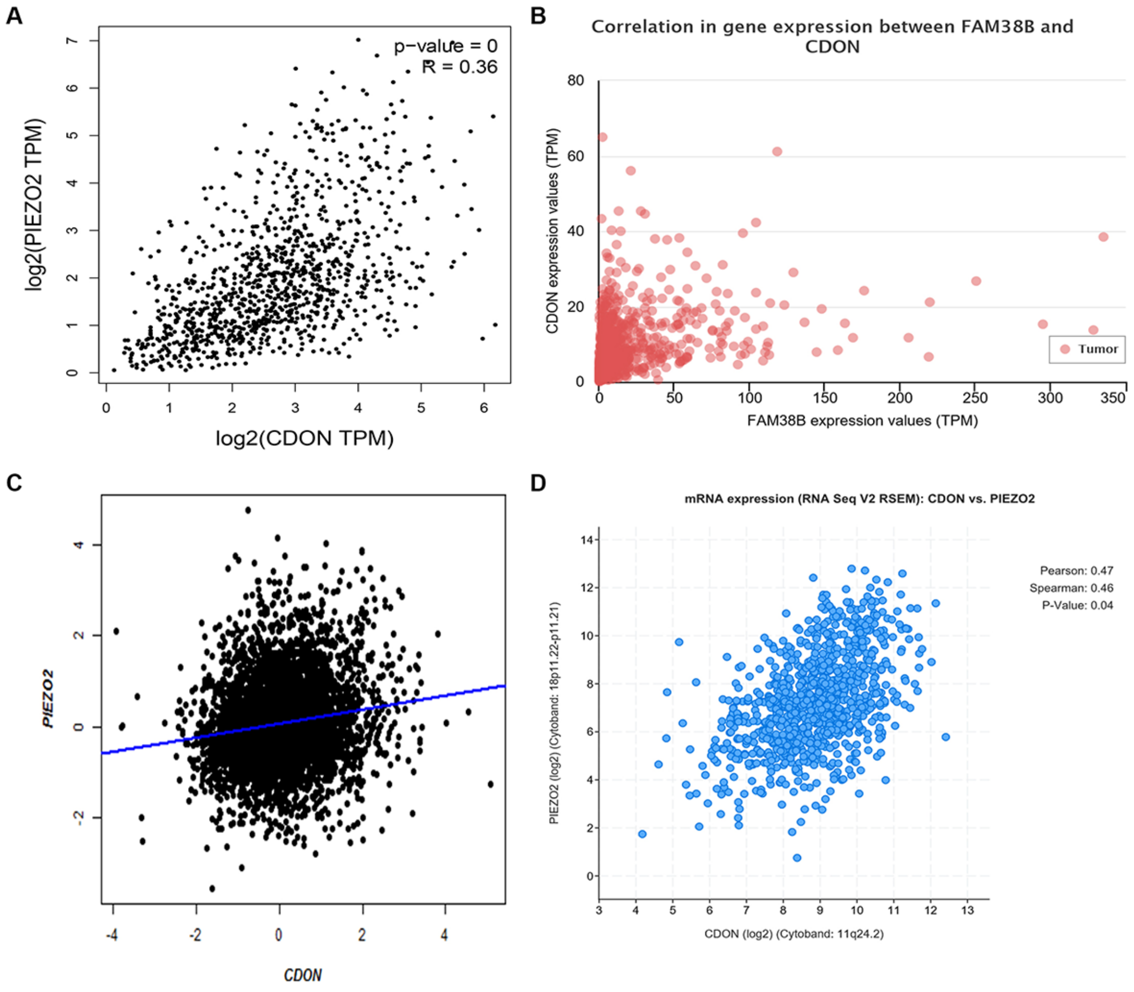

Figure S2. Correlation of PIEZO2 and CDON expression in breast cancer analyzed by GEPIA (**A**), UALCAN (**B**), bc-GenExMiner (**C**) and cBioPortal (**D**).

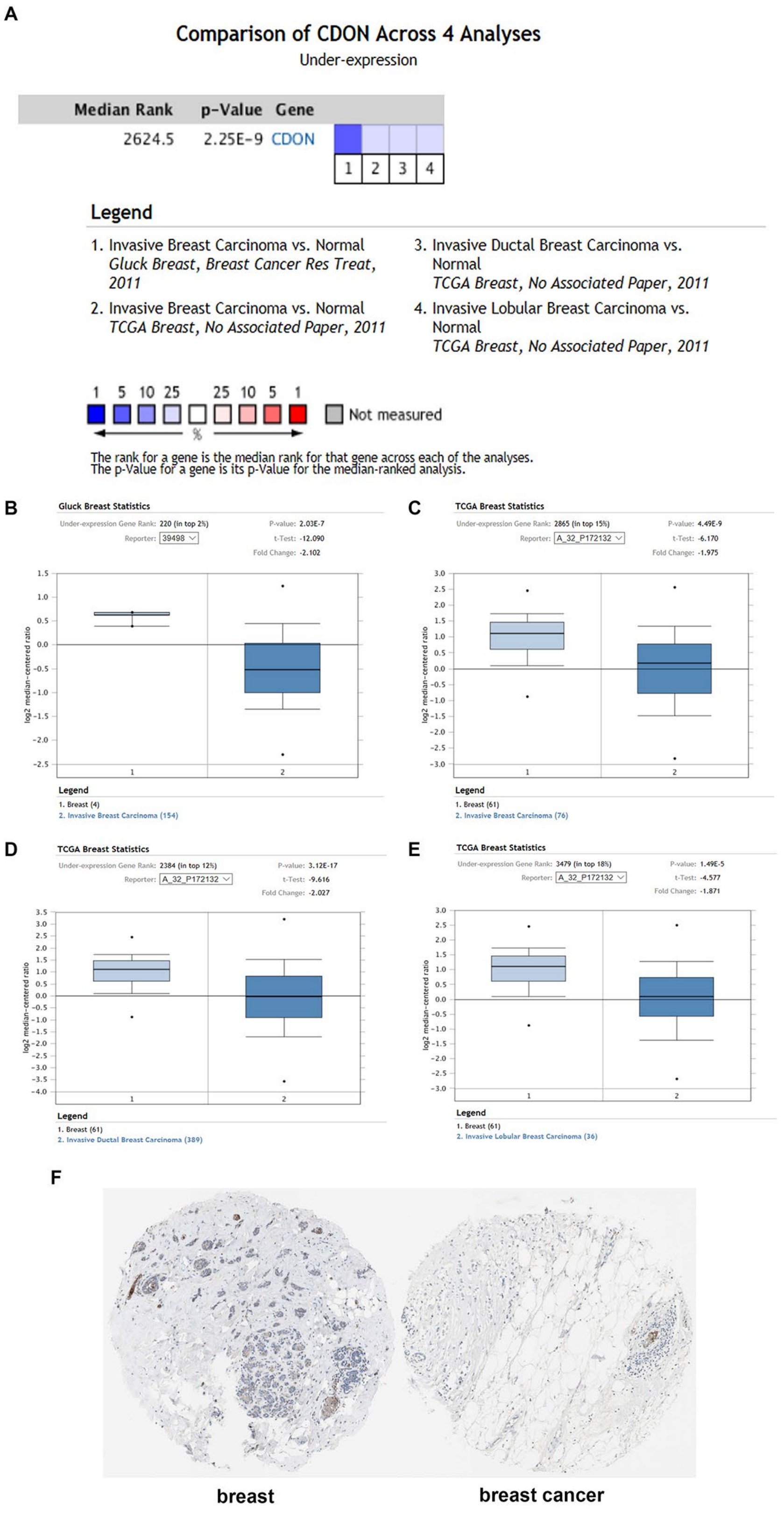

Figure S3. Expression of CDON in breast cancer. (**A**) Comprehensive meta-analysis of CDON expression across 4 datasets from Oncomine database; (**B-E**) comparison of CDON expression between breast cancer tissues and normal breast tissues in each of the four datasets; (**F**) CDON protein expression level in breast cancer tissue and normal breast tissue was analyzed using immunohistochemical staining from HumanProteinAtlas database.

Table S2. Sequences of primers and siRNAs used in this study.

qRT-PCR primers		Sequences
PIEZO2	Forward primer	ATGGCCTCAGAAGTGGTGTG
PIEZO2	Reverse primer	ATGTCCTTGCATCGTCGTTTT
CDON	Forward primer	TATCTCATGGCTGCATAACGGA
CDON	Reverse primer	TGTCAGAGTCCCCTGATGAATC
GAPDH	Forward primer	AATGGACAACTGGTCGTGGAC
GAPDH	Reverse primer	CCCTCCAGGGGATCTGTTTG
siRNA		
siRNA-PIEZO2	Sense	5’-UAAUGUAAUUGGUCAACGA-3’
	Antisense	5’-UCGUUGACCAAUUACAUUA-3’
siRNA-CDON	Sense	5’-GGAUCUUGGACCCUUAUGU-3’
	Antisense	5’-ACAUAAGGGUCCAAGAUCC-3’
